# Induced Pluripotent Stem Cell-Derived Exosomes Promote Peripheral Nerve Regeneration in a Rat Sciatic Nerve Crush Injury Model: A Safety and Efficacy Study

**DOI:** 10.3390/cells14070529

**Published:** 2025-04-02

**Authors:** Fatima Aldali, Yujie Yang, Chunchu Deng, Xiangling Li, Xiaojian Cao, Jia Xu, Yajie Li, Jianlin Ding, Hong Chen

**Affiliations:** 1 Department of Rehabilitation Medicine, Tongji Hospital, Tongji Medical College, Huazhong University of Science and Technology, Wuhan 430030, China; fatima.aldali12@gmail.com (F.A.); m202276342@hust.edu.cn (Y.Y.); deng_c@tjh.tjmu.edu.cn (C.D.); 13508285725@163.com (X.L.); zephyrus@hust.edu.cn (X.C.); jiaxuz0604@hust.edu.cn (J.X.); 2018tj5463@hust.edu.cn (Y.L.); 2 Stem Cell Research Center, Tongji Hospital, Tongji Medical College, Huazhong University of Science and Technology, Wuhan 430000, China; 3 Department of Gynecology & Obstetrics, Tongji Hospital, Tongji Medical College, Huazhong University of Science and Technology, Wuhan 430030, China; dingjl830258@tjh.tjmu.edu.cn; 4 Hubei Key Laboratory of Neural Injury and Functional Reconstruction, Huazhong University of Science and Technology, Wuhan 430030, China

**Keywords:** induced pluripotent stem cell, exosome, safety, nerve regeneration, Schwann cells peripheral nerve injury

## Abstract

Peripheral nerve injury (PNI) remains a significant clinical challenge, often leading to long-term functional impairment. Despite advances in therapies, current repair strategies offer unsatisfactory clinical outcomes. Exosomes derived from induced pluripotent stem cells (iPSC-Exos) have emerged as a promising therapeutic approach in regenerative medicine. This study assesses the efficacy and safety of iPSC-Exos in a rat model of sciatic nerve crush injury. Briefly, iPSCs were generated from peripheral blood mononuclear cells (PBMCs) of healthy donors using Sendai virus vectors and validated for pluripotency. iPSC-Exos were characterized and injected at the injury site. Functional recovery was assessed through gait analysis, grip strength, and pain response. Histological and molecular analyses were used to examine axonal regeneration, myelination, Schwann cell (SC) activation, angiogenesis, and changes in gene expression. iPSC-Exos were efficiently internalized by SC, promoting their proliferation. No adverse effects were observed between groups on body weight, organ histology, or hematological parameters. iPSC-Exos injection significantly enhanced nerve regeneration, muscle preservation, and vascularization, with RNA sequencing revealing activation of PI3K-AKT and focal adhesion pathways. These findings support iPSC-Exos as a safe and effective non-cell-based therapy for PNIs, highlighting their potential for clinical applications in regenerative medicine.

## 1. Introduction

“Peripheral-nerve injury” (PNI) is damage to the peripheral nerve trunk or branches outside the brain and spinal cord from direct or indirect external trauma, leading to clinical symptoms such as motor and sensory dysfunction and may result in activity loss, pain, depression, and, reduced quality of life [1,2,3]. PNI is a global issue, with a yearly incidence rate of about 13/100,000 to 23/100,000 in developed countries [4,5]. PNI is known to have a greater capacity for repair and regeneration compared to the central nervous system (CNS), which is primarily attributed to the characteristics of functional environments within each system [6,7]. Treatments for PNI include surgical intervention, nerve transplantation, and a range of pharmacological and physical therapies [7]. Autologous nerve transplantation is regarded as the gold standard for repairing injury to the peripheral nerve [8]. However, even under ideal conditions, autologous nerve transplantation fails to restore impaired motor and sensory functions [9].

Additionally, it shows notable limitations, including extended surgical duration, limited donor sites for repairing long or multiple nerve defects, potential complications at the donor site such as painful neuroma, scarring, and sensory deficits, and high financial costs [10]. Several approaches for PNI repair have recently been developed, showing promising outcomes in restoring the continuity of damaged neuroanatomy [7]. However, functional recovery is still challenging and unsatisfactory, especially in cases of severely injured peripheral nerves [11,12]. This particular situation has prompted the use of stem cell-based therapies to improve the function of peripheral nerve-related cells under injury and pathological conditions.

Mesenchymal stem cells (MSCs) are multipotent stem cells that may be derived from various tissues, including bone marrow, the umbilical cord, adipose tissue, and embryonic stem cells [13,14]. The emerging field of mesenchymal stem cell therapies holds promise for enhancing nerve regeneration and function [15]. Numerous studies have revealed that transplantation of MSCs on the site of peripheral nerve injury increased axonal regeneration and remyelination and promoted vascularization and angiogenesis [16,17,18,19]. Although the relatively easy isolation and availability of MSCs and their promising potential to differentiate into Schwann cell-like cells and promote axonal regeneration [20,21], significant challenges remain. However, one significant issue is maintaining the viability and vitality of these cells [22]. In addition, after in vivo transplantation, MSCs may differentiate into undesired cell lineages to peripheral nerves. These dysfunctions, transformations, and safety are all associated with transplanted stem cells, offering a potential alternative to non-cell-based tissue regeneration therapies [23,24].

Induced pluripotent stem cells (iPSCs) technology includes reprogramming terminally differentiated somatic cells into pluripotent stem cells by introducing particular transcription factors [25]. This reprogramming reverses differentiation, restoring the cells to a pluripotent state or forming embryonic stem cell lines [26]. Unlike traditional embryonic stem cells raise ethical issues, iPSC technology eliminates these issues since iPSC technology does not require embryonic cells or oocytes, thus avoiding ethical issues [27]. Moreover, iPSCs technology can produce patient-specific stem cells from the patient’s somatic cells, significantly reducing the likelihood of immune rejection [28]. Thus, the emergence of iPSCs has sparked significant interest in stem cell research, epigenetics, and biomedical sciences [29,30], providing new insights into the regulatory mechanisms of pluripotency and bringing stem cell and clinical disease treatment closer together. iPSCs hold cardinal potential for cell replacement therapy, drug screening, pathogenesis research, and neurological disease therapy [31]. Many researchers suggested that the therapeutic effects of MSCs arise mainly from the production of extracellular vesicles (Evs) rather than direct differentiation into functional neural cells [32]. As a result, mesenchymal stem cell-derived extracellular vesicles (MSCs-EVs) therapeutic applications have emerged as a promising cell-free therapy for PNI repair [33]. EVs are a category of heterogeneous nanoparticles conventionally clarified by their size and biogenesis [34]. Exosomes (Exos), a subtype of Evs, are nanosized extracellular vesicles of endosomal origin, typically with a diameter of ~30–150 nm comprising a lipid bilayer that carries bioactive molecules such as proteins, lipids, DNA, mRNA, miRNA, ncRNA, and circRNA [35,36] that regulate cellular communication and enhance peripheral nerve regeneration [33,37,38]. MSC-Exos can promote Schwann cell (SC) proliferation, axonal regeneration, and angiogenesis by providing growth factors (NGF, BDNF, VEGF), anti-inflammatory cytokines (IL-10, TGF-β), and miRNAs (miR-21, miR-126, miR-132) [39,40,41,42]. Furthermore, MSC-Exos have been found to activate essential signaling pathways, including PI3K/AKT, ERK/MAPK, TLR, NF-κB, MAPK, and STAT, which are critical for neuroprotection, regenerative ability, and regulating of neuroinflammation [40,43]. Numerous studies called attention to the importance of thoroughly analyzing exosomes, including their lipid, protein, and nucleic acid content, alongside conducting animal toxicology studies to identify potential safety concerns [44].

Exosomes hold great promise for treating different diseases, but it is essential to confirm their safety through comprehensive preclinical experiments before expanding to clinical applications.

Despite the growing interest in exosome-based therapeutics, research on iPSC-Exos remains limited. Only a few studies have examined their potential for treating stroke, spinal cord injury, and Alzheimer’s disease, indicating significant gaps in our understanding of their regeneration mechanisms and therapeutic applications [45,46,47]. This reveals the critical need for further investigation into iPSC-Exos, particularly in peripheral nerve regeneration, to maximize their potential for clinical translation. Therefore, in this study, we investigated iPSC-Exos safety and the efficiency of local injection for peripheral nerve regeneration in a rat sciatic nerve crush injury model.

## 2. Materials and Methods

### 2.1. Blood Sample Collection

Blood samples were collected from healthy donors (25-year-old males) using anticoagulant tubes, with 4~5 mL of peripheral blood obtained per sample. Informed consent was obtained from participants. The Ethics Committee of Tongji Hospital, Tongji Medical College, Huazhong University of Science and Technology approved the study. The study was approved on 1 November 2021 (Approval Number: TJ-IRB20211118).

### 2.2. Peripheral Blood Mononuclear Cells (PBMCs)

Ficoll-Paque PREMIUM was prepared in a separate 15-mL tube. A dilution of 4 mL of peripheral blood with an equal volume of DPBS at a 1:1 ratio was made. The diluted blood was then carefully layered onto the Ficoll solution, allowing for the formation of distinct layers upon centrifugation. The upper yellow layer contained platelet-rich plasma, the bottom clear layer was the Ficoll solution, and a thin white layer in between represented the PBMCs. The PBMC layer was collected gently after centrifugation without disturbing the underlying Ficoll layer. To further process the PBMCs, they were diluted and centrifuged twice by adding 5 mL of DPBS (Gibco, Grand Island, NY, USA) After centrifugation, the supernatant was discarded, and the PBMCs were resuspended in a T cell medium to achieve a concentration of 5 × 10^5^ cells/mL. Finally, the resuspended cells were seeded into 24-well plates that had been coated with anti-CD3 monoclonal antibodies.

### 2.3. Reprogramming PBMCs to iPSC (Feeder-Free)

On Day 5 of culture, PBMCs were selectively stimulated with anti-CD3 monoclonal antibody and IL-2, selectively proliferated T cells, and increased the proportion of T cells in the cultured PBMCs, based on the protocol described [48]. Then CD3+ T cells were transduced with Sendai virus (SeV) expressing human Oct3/4, Sox2, KLlf4, and c-Myc at a multiplicity of infection (MOI) of 20. Excess-infected PBMCs can be used as a positive control for assessing the removal of SeV by PCR. At around day 14, after the transduction of SeV, iPSC colonies emerge on the Vitronectin-N (VTN-N) coated plates. After picking up and expanding the iPSC colonies, the Culture medium mTeSR™ Plus was changed daily. Passage and cryopreservation use ReLeSR™ (Stemcell Technologies, Vancouver, BC, Canada) and CryoStor^®^ CS10 (Stemcell Technologies, Vancouver, BC, Canada), respectively. Until the fifth generation of iPSC, the cells on the first day after passage were still cultured in an incubator at 37 °C. To better inactivate the SeV, we transferred these cells to a 39 °C incubator for 5 days the next day and then returned to 37 °C for further cultured. Then, after two passages, a certain amount of cells were used to extract RNA. The mature iPSC clones were continuously selected for passage, and the morphology and generation of iPSC were recorded until Passage 35.

### 2.4. iPSC Characterisation

The staining of Alkaline Phosphatase (ALP) and (Yeasen, Shanghai, China) the pluripotency markers of iPSCs (NANOG, OCT4, SOX2, and SSEA4) was conducted on induced cell colonies. We followed the guidelines provided in the Alkaline Phosphatase Stain Kit for the dyeing process. Immunofluorescence staining of the iPSC pluripotency markers was performed based on a previously established protocol [49]. Total RNA was extracted from iPSCs at passage 7 using the RNA Easy Fast animal tissue/cell total RNA extraction kit (TIANGEN, Beijing, China). PCR analysis was employed to detect the presence of Sendai virus vectors within the iPSC lines. For a positive control, excess-infected PBMCs were utilized. The PCR and agarose gel electrophoresis experiments were outsourced to Beijing Tsingke Biotechnology Co., Ltd. (Beijing, China). Karyotype analysis was delegated to the obstetrics laboratory at Tongji Hospital.

### 2.5. Extraction and Identification of iPSC-Exos

iPSCs were seeded at a density of 3 × 10^6^ cells per 75 cm^2^ in culture dishes; when cell growth confluence reached 80%, the fresh serum-free medium was replaced, and the supernatant was collected 24–36 h later, at 4 °C. Centrifugation was performed at 300× *g* for 10 min, at 2000× *g* for 10 min to remove dead cells, at 10,000× *g* for 30 min to remove cell debris, followed by filter the supernatant with a 0.22 μm filter to remove large particles, and at 100,000× *g* ultra-fast centrifugation for 70 min to collect Exosomes [50]. The iPSC-derived exosomes were washed with phosphate-buffered saline (PBS), and stored at −80 °C for later use [51].

### 2.6. Transmission Electron Microscopy (TEM)

Ten microliters of isolated iPSC-Exos were carefully pipetted onto a 200-mesh copper grid and allowed to adsorb for 5–10 min. The excess liquid was carefully removed with filter paper, and the grid was fixed with 2% paraformaldehyde for 2 min at room temperature. The samples were rinsed three times with double-distilled water (ddH_2_O) to remove the remaining fixative, excess stain was blotted, and the sample was air-dried. The grids were then stained with 10 µL of 2% uranyl acetate for 1 min at room temperature to enhance contrast. and visualized under a TEM (×25,000, magnification) (Hitachi HT7700, Tokyo, Japan).

### 2.7. Nanoparticle Tracking Analysis (NTA)

The exosomes were characterized using nanoparticle tracking analysis (NTA) (Particle Metrix, Meerbusch, Germany). The sample pool was thoroughly cleaned with deionized water, and the instrument was then calibrated with polystyrene microspheres (100 nm) (Thermo Fisher Scientific, Fremont, CA, USA). After calibration, the chamber was rinsed with 1× PBS. Exosome samples derived from iPSCs were diluted 2000-fold in 1× PBS and injected into the NTA analyzer. The NTA software (ZetaView^®^ Software v8.5) measured the nanoparticle concentration (particles/mL) as previously described [52].

### 2.8. Western Blot Analysis

Western Blot Analysis was conducted to determine the protein expression of exosome markers in iPSCs and iPSC-Exos. The protein concentrations of iPSC-Exos or iPSCs were determined using a BCA assay kit (Beyotime, Shanghai, China) according to the manufacturer’s instructions. iPSC-Exos or iPSCs were lysed with RIPA buffer (Beyotime, Shanghai, China) containing a protease inhibitor cocktail (Sigma, USA). Fifteen micrograms of iPSC-Exos and iPSCs protein were separated by 10% SDS-PAGE and transferred to polyvinylidene fluoride membranes (PVDF) (Millipore Corporation, Billerica, MA, USA). The membrane was blocked with 5% skim milk powder for one hour and then incubated overnight at 4 °C with primary antibodies following polyclonal antibodies: monoclonal anti-CD9, CD63, GAPDH, and TSG101 (Dalian Meilun Biotechnology Co., Ltd., Dalian, China), CD81, and Calnexin (Proteintech, Rosemont, IL, USA). (Secondary antibodies (Signalway Antibody, College Park, MD, USA). were incubated with membranes for 1 h at 4 °C in the dark. Immunoreactive protein bands were visualized by adding g ECL reagent (Tanon, Shanghai, China) to the membranes, and the ChemiDoc MP imaging system (Bio-Rad, Hercules, CA, USA) was applied [53].

### 2.9. Exosome Labeling and Uptake Assay

The purified exosome was labeled with red fluorescence dye (PKH26) to assess their internalization in SCs following the manufacturer’s protocol (Sigma-Aldrich, St. Louis, MO, USA). Briefly, exosome pellets were resuspended in Diluent C. To prepare the labeling solution, 10 μL of PKH26 dye was mixed with 500 μL of Diluent C in an Eppendorf tube. The exosome suspension was combined with the dye mixture and incubated for 5 min. The reaction was terminated by adding 1% bovine serum albumin (BSA) in equal volume. To remove excess dye, the labeled exosomes were subjected to ultracentrifugation at 100,000× *g* for 90 min at 4 °C, then washed with PBS, and ultracentrifuged again for collection. The SC 96 cell line was obtained from Procell (Procell, Wuhan, China) for this study. SCs were incubated with PKH26-labeled exosomes (20 μg/mL) in a 35-mm confocal dish for 24 h. The cells were rinsed with PBS, fixed with 4% paraformaldehyde for 15 min, and then incubated with SF488 Phalloidin (1:200, Solarbio, Beijing, China) at 37 °C for 30 min. Nuclei were dyed with Hochest (1:1000, Invitrogen, Carlsbad, CA, USA for 8 min. The images were obtained using the confocal microscope and observed by laser-scanning confocal microscope (LSCM, FV1000, Olympus, Tokyo, Japan).

### 2.10. Cell Viability Assay

The proliferation of SCs was determined using Cell Counting Kit-8 (CCK-8, Beyotime, Shanghai, China). RSCs were seeded in 96-well plates at a density of 4 × 10^3^ cells per well overnight. Then, the cells were treated with iPSC-EXO at various concentrations (0, 1, 10, 50, and 100 μg/mL) for 24 h. After the incubation, 10 μL of CCK-8 solution was added to each well and incubated at 37 °C in humidified air containing 5% CO_2_ for 2 h. The cell proliferation was measured using a full-wavelength microplate reader at 450 nm.

### 2.11. Animals

This study followed the ARRIVE guidelines 2.0. Five-week-old male Sprague-Dawley (SD), weight 204 ± 14 g, was obtained from the Experimental Animal Center at Tongji Hospital, Tongji Medical College, Huazhong University of Science and Technology. Two rats per cage were housed in a standard cage in a free specific pathogen-free (SPF) room under standardized conditions (21 °C, 55% humidity, and a 12-h light-dark cycle) with free access to libitum food and water. All operations followed the China Laboratory Animal Care and Use Guidelines and were authorized by the animal care and use committee of Tongji Hospital, Tongji Medical College of Huazhong University of Science and Technology (Registration number. T1-2024-06-069). Rats’ body weights and behavior tests were recorded one- day preoperatively and postoperatively on days 7, 14, 21, and 28 post-surgery.

### 2.12. Nerve Crush Injury and Exosomes Implantation

Forty-eight SD rats were anesthetized in an induction chamber with 3.5% isoflurane and 100% oxygen. Following induction, rats were placed in the prone position, and anesthesia was maintained with 1.5–2% isoflurane and 100% oxygen administered through an anesthetic mask. The depth of anesthesia was assessed by monitoring the breathing rate and adjusted during surgery accordingly, body temperature was maintained at 37 °C using a heat pad. Rats were randomly divided into three groups: (a) sham group n = 12, (b) Model + PBS group n = 18, (c) Model + iPSC-Exos group n =18. A 2 cm incision along the lateral aspect of the hind limb, spreading the gluteal muscles apart. The sciatic nerve was exposed and crushed using an ultra-fine hemostat (Fine Science Tools, Heidelberg, Germany; Cat. No. 13020-12) for 60 s until the nerve became translucent, covering an area of approximately 3–4 mm^2^, a 9–0 nylon epineural suture was used to mark the area for later identification [54]. This procedure fully transected the sciatic nerve axon while leaving the epineurium intact, as reported previously [54]. Rats in the iPSC-Exos group received an injection containing 10 μL of 1.1 × 10^11^ particles/mL of iPSC-Exos under the epineurium at both the proximal and distal ends of the crushed nerve site using a micro syringe (Hamilton, Reno, NV, USA). The syringe was pushed continuously for 2 min under the epineurium (Figure 3B). PBS group rats received 10 µL of PBS at the same sites. While rats in the sham group underwent the same procedure, and the nerve was left intact. The muscle layers and skin were sutured with 4/0 silk sutures. After the operation, a weekly rat body weight, footprint analysis, grip strength, von fery filament, and mechanical pain were performed for 4 weeks. Rats were sacrificed on day 7 to conduct qPCR and RNA-seq to investigate gene expressions of SC activity, inflammation, and axonal regeneration and to evaluate possible pathways involved in exosome-mediated nerve regeneration. On day 14, histological examinations of remyelination, axonal regeneration, SC proliferation, and vascularization were conducted. On day 28, the final histological evaluations for remyelination, axonal regeneration, and vascularization analyses (Figure 3A). Rats were anesthetized with isoflurane (5% induction, 1.5–2% maintenance) and euthanized either transcardial perfusion for histological analysis or cervical dislocation for molecular studies. Tissue samples were immediately frozen for qPCR/RNA-seq or fixed for histology.

### 2.13. Reverse Transcription-Quantitative Polymerase Chain Reaction (RT-qPCR)

Frozen nerve tissue was homogenized in liquid nitrogen. Total cellular RNA was extracted from nerve tissue using a Fast Pure Tissue Total RNA Isolation Kit (RC101, Vazyme, Nanjing, China). cDNA synthesis was performed using HiScript^®^III RT SuperMix (R323–01, Vazyme, Nanjing, China) following the manufacturer’s instructions. The relative mRNA levels were measured using ChamQ Universal SYBR qPCR Master Mix (Q711–02; Vazyme, Nanjing China). The relative gene expression was normalized to GAPDH and quantified using the 2−^ΔΔCt^ method. All samples were examined in triplicate, and relevant primer sequences are listed in (Table 1).

### 2.14. Rat Sciatic Functional Index (SFI) Analysis

To quantify motor function recovery, we performed SFI analysis, a standard measure of locomotor performance in nerve injury models. Footprint analysis was conducted at 1 day preoperative and postoperative, on days 1, 7, 14, 21, and 28 following iPSC-Exos injection; rats were held by the chest while their hind limbs were coated with stamping ink. Following acclimatization period trials, the rats walked through a tunnel 8.2 cm wide and 42 cm long with a dark shelter at the end, leaving their inked footprints on a paper strip [55,56]. At least three footprint pairs were analyzed per animal from each group (n = 6). The SFI was calculated as the following formula [57].

SFI = 109.5 × (ETS − NTS)/NTS − 38.3 × (EPL − NPL)/NPL + 13.3 × (EIT − NIT)/NIT − 8.8

where E and N represent experimental and normal, respectively; the entire plantar length (PL) is the distance from the heel to the third toe; the toe spread (TS) is the distance from the first to the fifth toe; and the intermediary toe spread (IT) is the distance from the second to the fourth toe. SFI indicates the recovery of the sciatic nerve. An SFI of −100 represents a complete loss of function, while a number around 0 suggests an efficient nerve function and recovery. Since sciatic nerve injury affects muscle strength, we subsequently measured grip strength to measure functional motor recovery.

### 2.15. Grip Strength Measurement

Grip strength was measured with a grip strength meter grip strength meter (TSE Systems, Bad Homburg, Hesse, German) one day preoperatively and postoperatively on days 7, 14, 21, and 28 [58]. A 6 cm-wide grip with a sensor and amplifier was used. Four measurements were recorded at 10-s intervals, allowing for a rest period between each measurement. The average tension force for each group was determined by averaging these values for each group (n = 6). During the test, one handheld the animal’s shoulders, and the other supported its lower body and legs. The grip was demonstrated by gently placing one foot over it and allowing the animal to grab it. The animal was then steadily tugged by its tail until it Released off the grasp. Further, pain hypersensitivity is a common result of nerve injury; next, we assessed mechanical pain thresholds to determine sensory recovery.

### 2.16. Assessment of Nociceptive Responses

Rats were adopted for 30 min in an elevated cage with wire mesh bottoms in the animal behavior testing room. The Von Frey mechanical pain threshold was measured using the model 2390 Electric von Frey Aesthesiometer (IITC Life Science, Woodland Hills, CA, USA) to assess rats’ paw mechanical withdrawal thresholds (PWTs) [59]. PWTs were measured one day preoperatively and postoperatively on days 7, 14, 21, and 28 postoperative in each group (n = 5). With an appropriate probe that applied incremental forces on the ventral surface of the hind paw. A paw withdrawal response response, such as brisk lifting and paw withdrawal, was documented with a digital meter. The cut-off pressure was set to 400 g to avoid tissue damage. Paw withdrawal pressure was measured four times for both hind paws of each animal, and the average of the four readings was used to calculate the percentage of the operated versus the contralateral intact hind paw, with 10-min intervals for each reading.

### 2.17. Systemic and Local Biocompatibility of iPSC-Exos Following Nerve Injury

To evaluate potential toxicity, we measured rat body weight weekly, as significant weight loss could indicate adverse effects of iPSC-Exos treatment. A complete blood count (CBC) was performed preoperation one day and again one week after postoperation to evaluate potential blood parameter deviations for toxicity evaluation. Blood was collected under general anesthesia using isoflurane. The tail vein was disinfected with Poldine, and 1 mL of blood was drawn using a sterile 1 mL syringe from each rat (n = 6 per group). The blood was transferred to 2 mL BD EDTA tubes for each group. When multiple samples were collected, an icebox was used to prevent hemolysis. Blood samples were analyzed immediately after sample collection using a fully automated blood cell analyzer (BC-6600 Mindray, Shenzhen, China) [60]. After the behavioral tests were completed over 4 weeks, rats were deeply anesthetized with terminal anesthesia with an overdose of isoflurane (5% induction, 1.5–2% maintenance), and perfused transcardially with 0.9% saline (0.9% NaCl), followed by perfusion with 4% paraformaldehyde (PFA) in PBS. Following, the kidneys, hearts, lungs, livers, and spleens (n = 6) in all groups of organs were weighed using a digital scale to assess any weight changes associated with iPSC-Exos toxicity

### 2.18. Muscle Wet Weight, and Histological Examination of the Gastrocnemius Muscle

After the behavioral tests were completed over 4 weeks, rats were deeply anesthetized with terminal anesthesia with an overdose of isoflurane (5% induction, 1.5–2% maintenance), and perfused transcardially with 0.9% saline (0.9% NaCl), followed by perfusion with 4% paraformaldehyde (PFA) in PBS. Following, the kidneys, hearts, lungs, livers, and spleens (n = 6) in all groups of organs were weighed using a digital scale to assess any weight changes associated with iPSC-Exos toxicity. The gastrocnemius muscles on both sides of each rat were dissected for each group (n = 8), photographed, and weighed using an electronic scale. Rats’ gastrocnemius muscles were randomly selected from each group and fixed in 4% paraformaldehyde for 24 h and embedded in paraffin. Transverse sections of the muscle, four 4 μm thick, were prepared and stained with a Masson staining kit (BIOSSCI Biotech, Beijing, China) according to the manufacturer’s instructions. Images captured microscopically (Olympus, CX-31, Tokyo, Japan). Five muscles from each group were randomly selected, and four random visual fields were selected from each sample. The mean cross-sectional area of muscle fiber was analyzed with software (ImageJ, Version 1.53t, NIH, USA).

### 2.19. TEM, H&E Staining

Rats were euthanized with an overdose of isoflurane and perfused transcardially with 0.9% saline (0.9% NaCl), and nerve tissues were carefully excised and fixed with 2.5% glutaraldehyde at 4 °C for two hours. The tissues were then stained with 1% osmium acid, dehydrated with an acetone gradient, and embedded in Epon812 epoxy resin. They were cut into semithin sections at (0.7 µm) and ultrathin sections at 70 nm, and ultrathin sections were stained with 3% lead citrate- uranyl acetate. The samples were visualized and imaged using a transmission electron microscope (TEM) (HT7700, Hitachi, Tokyo, Japan). The thickness of axons and myelinated nerve fibers was measured using ImageJ software. H&E staining procedure briefly: the rats’ nerves in each group were harvested and fixed in 4% paraformaldehyde for 2 h and embedded in paraffin. The nerves were cut into 4 μm thickness transverse sections and stained with a staining kit (BIOSSCI Biotech, Beijing, China) according to the manufacturer’s instructions. Microscopic images were captured using an (Olympus Corporation, Tokyo, Japan). To examine the potential pathological effects of iPSC-Exosomes injection on key organs, including the liver, lungs, spleen, kidneys, and heart, visual and pathological analyses were performed (n = 6). These analyses focused on detecting abnormalities or inflammatory cell infiltration in tissue sections from the liver, heart, spleen, lungs, and kidneys.

### 2.20. Immunofluorescence Staining

The dissected nerve tissues were carefully excised and fixed in 4% paraformaldehyde overnight and cryoprotected in 10%, 20%, and 30% sucrose gradient and embedded in O.C.T. The tissues were sliced longitudinally and cross-section into a thickness of 10 μm in a cryostat. The iPSCs or nerve sections were incubated with 0.1% Triton X-100 for 20 min and incubated with 10% goat sealing serum for 50 min then incubated with antibodies as follows: The antibodies used for immunostaining were as follows: goat anti-NANOG antibody (1:50, AF1997, R&D Systems, Minneapolis, MN, USA) rabbit anti-OCT4 antibody (1:500, P0056, Millipore Sigma, Burlington, MA, USA) rabbit anti-SOX2 antibody (1:500, AB5603, Millipore Sigma, Burlington, MA USA), mouse anti-SSEA4 antibody (1:500, ab16287, Abcam, Cambridge, UK), mouse anti-S100 antibody (1:100, MA1-26621, Thermo Fisher Scientific, Waltham, MA, USA) rabbit anti-NF200 antibody (1:400, ab215903, Abcam, UK), rabbit anti- CD31 (1:400, ab215903, Abcam, Cambridge, UK) diluted in blocking solution at 4 C overnight. The next day, the secondary antibody was incubated for one hour with secondary antibodies Alexa 594 anti-mouse (1:1000, Jackson ImmunoResearch, West Grove, PA, USA) Alexa 594 anti-goat (1:1000, Invitrogen, Carlsbad, CA, USA), Alexa 594 anti-mouse (1:1000, Invitrogen, Carlsbad, CA, USA), Alexa 488 anti-rabbit (1:1000, A21206, Invitrogen, Carlsbad, CA, USA), for one hour, and nuclear staining was performed using 4′,6-diamidino-2-phenylindole DAPI (1:1000, Abcam, Cambridge, UK) or and Hoechst 33342 (1:1000, Invitrogen, Carlsbad, CA, USA) for 5 min. Images were captured by laser-scanning confocal microscope (LSCM, FV1000, Olympus). Mean fluorescence intensity was quantified using ImageJ (National Institutes of Health, USA). Corrected total fluorescence for each cryosection was calculated as the average fluorescence intensity of the selected area, adjusted by subtracting the background fluorescence based on the mean intensity of the selected region product and the mean fluorescence of the background. The total number of positive cell staining was calculated using ImageJ.

### 2.21. RNA-Seq Library Construction

On postoperative day 7 rats were deeply anesthetized with an overdose of isoflurane, the rats were sacrificed, and the sciatic nerve was dissected. The total RNA of the nerves was extracted and purified with the TRIzol reagent (Thermo Fisher Scientific, Waltham, MA, USA) following the manufacturer’s protocols. The quality and quantity of RNA were assessed using a Nanodrop spectrophotometer (Thermo Fisher Scientific, Waltham, MA, USA) and the Agilent Bioanalyzer 2100 system to ensure sample integrity. All RNA-seq data supporting this study are available online in the Gene Expression Omnibus (GEO) database under the accession number GSE286150. The data is scheduled to be made publicly available on 8 January 2026.

### 2.22. RNA-Seq Data Processing

The sequencing adaptors and low-quality bases were trimmed using Trimmomatic (version 0.38) to ensure a high-quality read [61]. High-quality paired-end reads were aligned to the reference genome using HISAT2 (version 2.0.5) [62]. The reads were subsequently mapped to individual genes, and the FPKM (Fragments Per Kilobase of transcript per Million mapped reads) of each gene was calculated based on gene length and mapped reads. Differential expression analysis between the two conditions was performed using the DESeq2 R package (version 1.16.1) [63]. Genes with a *p*-value ≤ 0.05 and an absolute log2 fold change (log2FC) ≥ 0.5 were identified as differentially expressed genes (DEGs). Gene Ontology (GO) pathway enrichment analysis and visualization were performed using the clusterProfiler R package [64].

### 2.23. Image Analysis

Immunofluorescence sections stained for NF200, S100, and CD31 were analyzed by capturing 3–5 fields of each rat and were viewed (20× magnification) to quantify the extent of regeneration. To evaluate nerve regeneration, we analyzed NF200 (axon count), S100 (Schwann cell positive area), and CD31 (fluorescence intensity). The mean thickness of myelinated nerve fibers and axons was also measured from 3 randomly selected areas within each TEM image. For muscle analysis, the mean cross-sectional area (CSA) and nerve-stained areas were determined by capturing 3–5 random fields of view (40× magnification) from the sections. Image analysis software (ImageJ) was used to perform cell counting and quantify early vascular regeneration, axonal outgrowth, and myelin regeneration.

### 2.24. Statistical Analysis

Statistical analysis was conducted using GraphPad Prism 9.0 software (San Diego, CA, USA). In comparisons between the two groups, a student’s t-test was employed, and A one-way ANOVA was performed to compare differences among three or more independent groups, followed by Tukey’s post hoc test. Data were expressed as Mean ± Standard Error of the Mean, meaningful significant differences and data were reported as mean values ± SEM. *p* < 0.05, *p* < 0.01, *p* < 0.001, and *p* < 0.0001 are shown as *, **, ***, and ****, respectively.

## 3. Results

### 3.1. Reprogramming and Characterization of iPSCs

**Figure 1:**
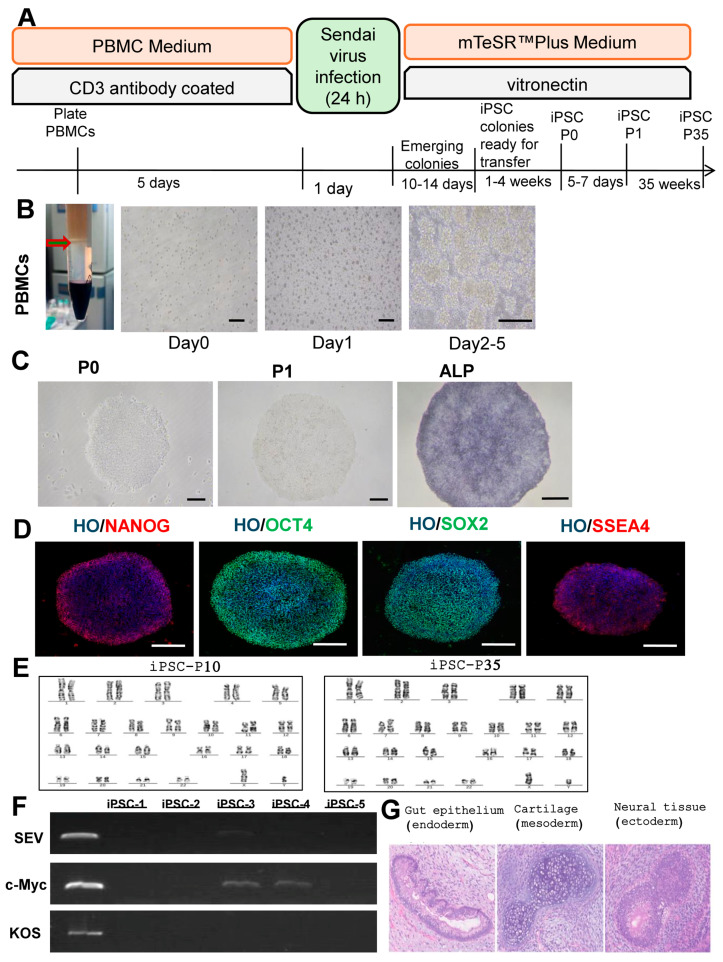
(**A**) Overview of the iPSC generation protocol. PBMCs are activated for 5 days with IL-2 and anti-CD3 antibodies and then transduced with SeV expressing human OCT3/4, SOX2, KLF4, and c-MYC. iPSC colonies emerge at 10–14 days after blood sampling. In the next month, mature iPSC clones were continuously picked for passage as P0, and the morphology of iPSC was recorded. Subsequently, it was passaged to the p35. (**B**) Isolation and activation of PBMCs. Morphology of PBMCs shortly after being seeded in the culture plate for 5 d. Scale bar, 0.2 mm. (**C**) ESC-like iPS colony after the first Passage, P1. And typical iPS colony after the second Passage, P2. ALP staining of iPSC colonies. Scale bar, 0.2 mm. (**D**) Immunofluorescence staining for pluripotency and markers (NANOG, OCT4, SOX2, SSEA4) in iPSCs. Hochest (HO) was used for nuclear staining. Scale bar, 100 μm. (**E**) We performed a temperature shift to remove SEV, c-Myc, and KOS vectors. These vectors had no obvious residues in the 5 random clones by PCR and agarose gel electrophoresis. (**F**) The karyotypes of normal males were verified at the 10th and 35th generations of iPSC, respectively. (**G**) Teratomas are derived from the iPSC-1 line. We observed the formation of various tissues from the three germ layers in NOD-SCID mice, such as neural tissue from ectoderm, gut epithelium from endoderm, and cartilage from mesoderm. Scale bar, 0.1 mm.

The process of generating iPSCs is outlined in Figure 1A. PBMCs, isolated from blood, are one of the most popular sources of somatic cells for iPSC generation, as shown in Figure 1B. iPSC colonies emerge around day 12 after the SeV transduction. By the third week, iPSC colonies had been selected and expanded. After the first passage (P1), the colonies exhibited an embryonic stem cell (ESC)-like morphology. By the second passage (P2), they exhibited typical iPSC colony characteristics. The iPSC colonies showed positive for alkaline phosphatase, a stem cell marker (Figure 1C). Furthermore, immunostaining confirmed the presence of important pluripotency markers such as NANOG, OCT4, SOX2, and SSEA4 (Figure 1D).

The remaining SeV-infected T cells as a positive control. We randomly selected five iPSC clones for RNA extraction and PCR detection at the P7. SEV and KOS had no residue in the five random clones, although c-Myc detected a small amount of residue in two iPSC clones. In the subsequent cell amplification culture, we continued to amplify and culture iPSC-1, and the rest was frozen for later use (Figure 1E). In the 10th and 35th generation of iPSC-1, we identified the karyotype and found that it was a normal male 46, XY karyotype without abnormal mutation (Figure 1F).

To evaluate the differentiation potential in vivo, iPSC cells were injected intra-muscularly into NOD-SCID mice, and then the resulting teratomas were histologically analyzed. The teratomas showed the formation of various tissues from the three germ layers, including neural tissue from the ectoderm, gut epithelium from the endoderm, and cartilage from the mesoderm (Figure 1G).

### 3.2. Characterization of iPSC-Exos

**Figure 2:**
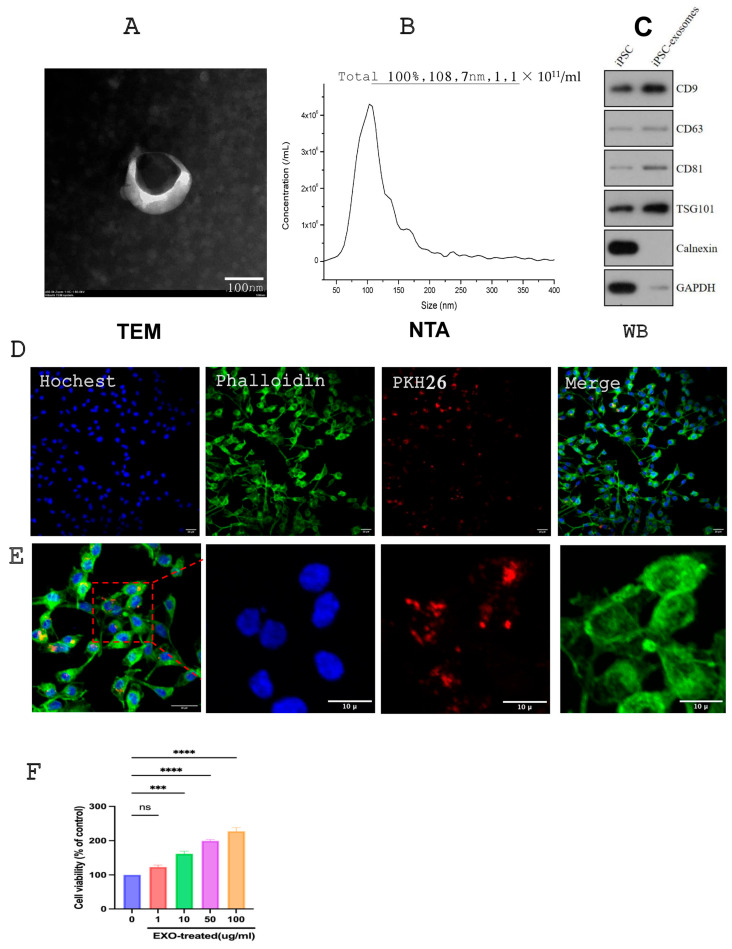
Characterization of iPSC-Exos. (**A**) Representative morphology of iPSC-Exos observed under transmission electron microscopy (TEM), scale bar: 100 nm. (**B**) Nanoparticle tracking analysis (NTA) of iPSC-Exos, showing particle size distribution and total concentration (particles/mL). (**C**) Western blot (WB) analysis of iPSC-Exos, confirming the presence of exosomal markers (Alix, TSG101, CD9, CD63, CD81) and GAPDH as a loading control, while Calnexin serves as a negative control. (**D**) Confocal microscopy images demonstrating the internalization of PKH26-labeled iPSC-Exos (red) into Schwann cells (SCs) via the endocytic pathway. Green fluorescence highlights SCs. Scale bars: 20 μm. (**E**) Magnified images confirming exosome uptake by Schwann cells. Scale bars: 10 μm. (**F**) Effect of iPSC-Exos on SC proliferation. SCs were treated with different concentrations of iPSC-Exos (0, 1, 10, 50, and 100 μg/mL) for 24 h, and cell viability was assessed using the CCK-8 assay. Data are presented as mean ± SD (n = 3). *** *p* < 0.001, **** *p* < 0.0001, ns: not significant.

TEM analysis verified the morphology of iPSC-derived Exosomes, revealing membrane-bound structures with a uniform size distribution (Figure 2A). Exosomes from the conditioned iPSC medium had a concentration of 1.1 × 10^11^ particles/mL, with an average size of 108.7 nm, indicating a relatively uniform particle population (NTA results). These findings show that most of the extracellular vesicles in this investigation were Exosomes (Figure 2B). Western blot analysis, according to the guidelines of the International Society for Extracellular Vesicles (ISEV) [65] verified the presence of Exosome markers, including tetraspanins (CD81, CD63, and CD9) and cytosolic protein (TGS101), while calnexin, an endoplasmic reticulum marker, was absent in iPSC-Exos but present in iPSCs (Figure 2C). These findings confirm that iPSC-Exos was successfully isolated for further experimental use. Furthermore, exosomes derived from iPSCs were labeled with PKH26, and labeled Exos were incubated with SCs and internalized by SCs. SCs were then stained with Phalloidin and Hochestfor nuclei and examined by immunofluorescence. The internalized exosomes displayed remarkable fluorescent signals (red), distributed around the nucleus (Figure 2D,E). To further investigate the effects of iPSC-Exos on SC proliferation, we treated SCs with iPSC-Exos at different concentrations (1, 10, 50, and 100 μg/mL) for 24 h. The CCK8 assay results showed that SCs treated with iPSC-EXO had higher absorbance values than those treated with PBS (Figure 2F). Data showed that iPSC-EXO enhanced SC proliferation in a concentration-dependent manner. According to these findings, 100 μg/mL of iPSC-Exos was selected as the experimental concentration for subsequent experiments and vivo experiments.

**Figure 3:**
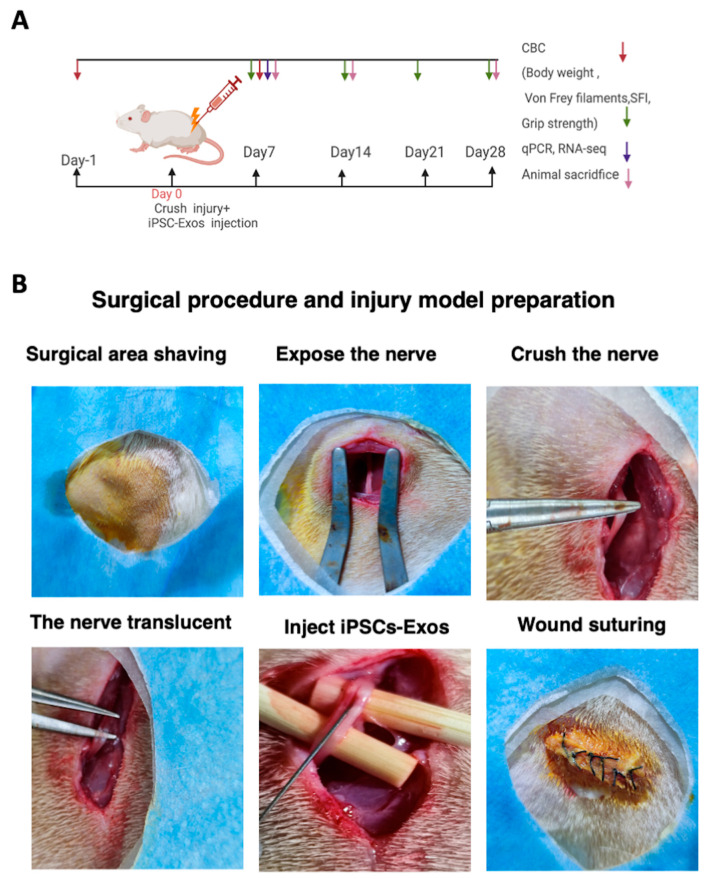
(**A**) Schematic illustration of the experimental design for sciatic nerve injury and subsequent functional recovery. Assessments, including complete blood count (CBC), behavioral tests (body weight, Von Frey filaments, Sciatic Function Index, and grip strength), qPCR, RNA-seq, and histological evaluations, were conducted at specific time points with animal sacrifice performed accordingly. (**B**) Representative images illustrating the surgical procedure and injury model preparation. The sciatic nerve was surgically exposed, crushed using FST Super Fine Hemostatic Forceps (13020-12, Heidelberg, Germany) for 60 s to ensure full injury of the nerve, and observed until it became translucent iPSC-Exos were injected at the injury site, followed by wound closure via suturing.

### 3.3. iPSC-Exosome Injection Is Safe, with No Evidence of Toxicity Observed

**Figure 4:**
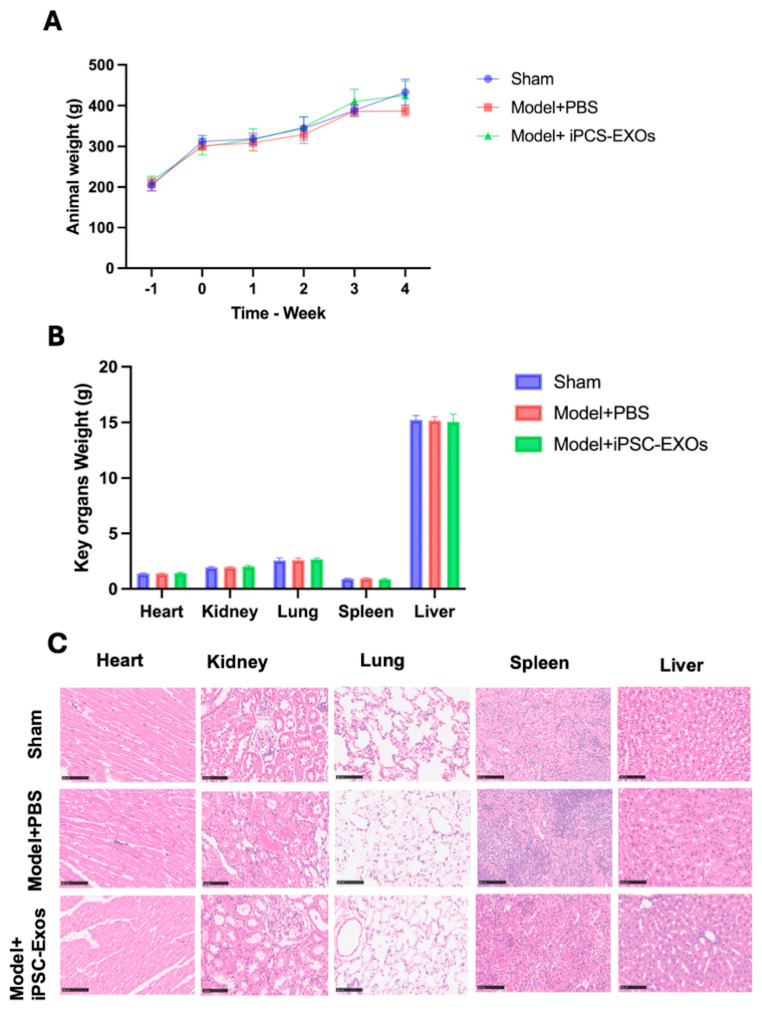
(**A**) Rats’ body weight was recorded one day preoperative and at 1, 2, 3, and 4 weeks postoperation. There were no statistically significant differences between the groups (n = 12 per group), indicating that the treatment with iPSC-Exos exhibited no effect on the overall growth of the rats. (**B**) To evaluate potential systemic toxicity, vital organ weights (liver, spleen, lung, kidney, and heart) were recorded four weeks after the sacrifice. There were no obvious differences between the Model + PBS, Model + iPSC-Exos, and Sham groups. (**C**) Representative hematoxylin and eosin (HE) at 4 weeks post the operation, representative hematoxylin and eosin (H&E)-stained sections of the liver, kidney, lung, spleen, and heart (n = 6 per group). No inflammatory infiltrates, structural anomalies, or signs of toxicity were observed. Scale bar of 100 μm. Data represent mean ± standard error and were analyzed using one-way ANOVA. Statistical analysis was performed using one-way ANOVA with Tukey’s multiple comparisons test, with significance set at * *p* < 0.05.

Behavioral assessments included observations of mobility, vocalization, and food intake, revealing no abnormalities between the groups. The multiple between-group comparisons with one-way ANOVA using Tuckey’s for multiple comparisons tests did not show any significant variation in animal weight between the groups at the baseline after the iPSC-Exos injection, (*p*  >  0.05) (Figure 4A). Furthermore, all the groups had similar growth patterns, as recorded by body weight, and none showed evidence of toxicity (Figure 4A). Routine blood parameters evaluation showed that all CBC parameters were within the normal range (Table 2).

No statistically significant treatment-related changes were observed in the hematological parameters across groups, indicating the safety of iPSC-Exos administration. While some values exceeded the reference ranges, similar variations were observed in both sham and PBS-treated groups, suggesting biological variability rather than a treatment effect. White blood cell counts remained within normal limits, while red blood cell and hemoglobin levels were slightly elevated in the iPSC-Exos group, but not significantly. Platelet counts were higher in the iPSC-Exos group but without abnormal platelet indices. Lymphocyte percentages increased while neutrophil percentages decreased in the iPSC-Exos group, suggesting a potential immunomodulatory effect. No significant deviations were noted for eosinophils and basophils. These findings confirm that iPSC-Exos does not induce systemic hematological toxicity (Table 2). An assessment of organ weights for key organs showed no significant differences between groups (Figure 4B), further confirming the safety of the iPSC-Exo treatment. Histological analysis at week 4 post-injury revealed normal organ morphology in the lungs, kidneys, heart, spleen, and liver, which indicates that iPSC-Exosome injections are safe and non-toxic to these organs (Figure 4C).

Table 2 Shows means with SD for different complete blood count parameters, assessed at week one post-iPSC-Exos injection to evaluate toxicity. WBC: white blood cell, RBC: red blood cells; HGB: hemoglobin; HCT: hematocrit; MCV: mean corpuscular volume; MCH: mean corpuscular hemoglobin; RDW: Red Cell Distribution Width-Standard Deviation; PLCR: Platelet Large Cell Ratio; PDW: platelet distribution width; MPV: mean platelet volume; PCT: plateletcrit, #; count, %: percent, TAC.

### 3.4. iPSC-Exos Upregulates the Expression of Genes Related to Nerve Regeneration

**Figure 5:**
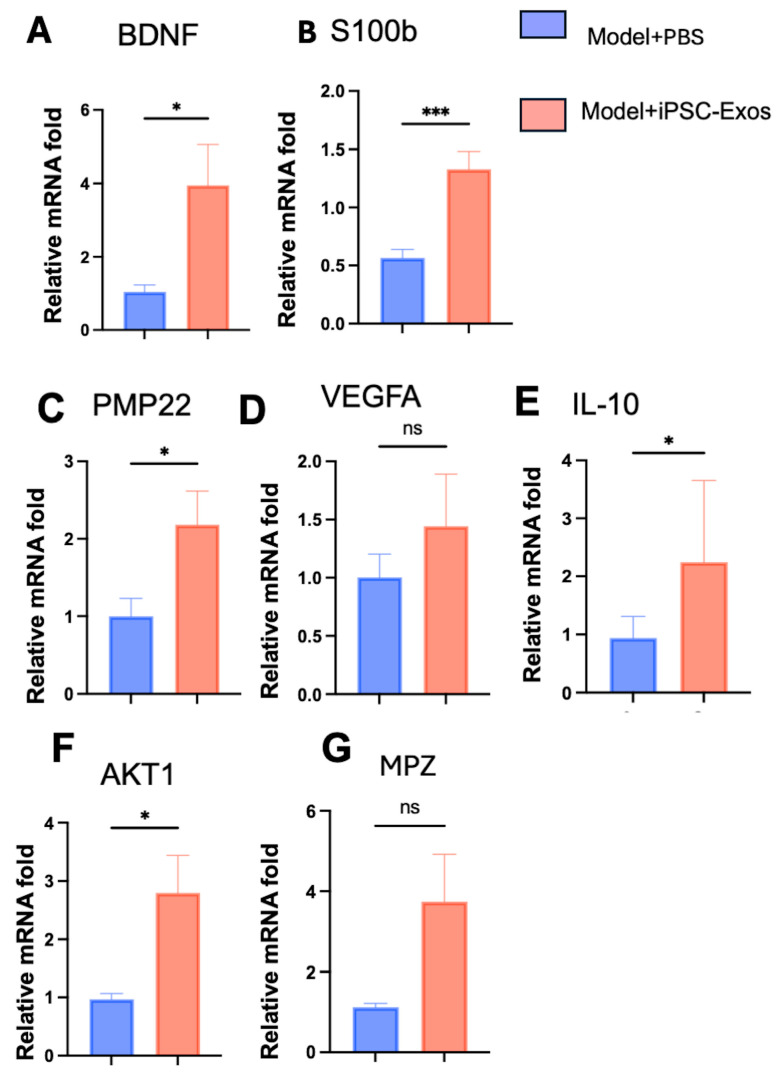
Gene expression analysis of nerve regeneration markers in PBS and iPSC-Exos treated group at 7 days postoperatively. (**A**) *BDNF*, (**B**) *S100β*, (**C**) *PMP22*, (**D**) *VEGFA*, (**E**) *IL-10*, (**F**) *AKT1,* and (**G**) *MPZ* mRNA levels were analyzed. *BDNF*, *S100β*, *PMP22*, *IL-10*, and *AKT1* were significantly upregulated in the iPSC-Exos group, indicating enhanced nerve regeneration, SC activation, myelination, and anti-inflammatory effects. *VEGFA* and *MPZ* showed an increasing trend but were not statistically significant. All results were normalized to *GAPDH.* Data were represented as mean ± standard error and were analyzed using a *t*-test. * *p* < 0.05, *** *p* < 0.001, ns: not significant.

To determine if iPSC-Exos-induced nerve regeneration involves the regulation of specific gene expressions, nerve tissue samples were collected from rats seven days after SNI. *The brain-derived neurotrophic factor (BDNF), peripheral myelin protein (PMP22), vascular endothelial growth factor A (VEGFA), interleukin-10 (IL-10), AKT serine/threonine kinase 1 (AKT1), S100 calcium-binding protein B (S100B), and myelin protein zero (MPZ)* expression levels were determined using qRT-PCR. The iPSC-Exos treated group revealed significantly higher expression levels of genes as mentioned above, compared to the PBS group. iPSC-Exos effectively promoted the expression of mature-type myelin-associated protein genes such as *PMP22 and MPZ*, critical to promoting remyelination and restoring nerve function (Figure 5C,G). In addition, iPSC-Exos upregulates *IL-10* and reduces inflammation, creating a conducive environment for repair (Figure 5E). iPSC-Exos group revealed up-regulation of *VEGFA*-enhanced angiogenesis with no significance compared to the PBS group. *BDNF* enhances Sc survival, axonal growth, and regeneration after PNI (Figure 5A). *S100B* and *AKT1* promote cell survival and axonal regeneration, ensuring effective nerve regeneration and functional recovery, these results indicate an association between these factors and the observed regeneration benefits.

### 3.5. iPSC-Exos Promotes Functional Recovery and Attenuates Mechanical Pain Following

**Figure 6:**
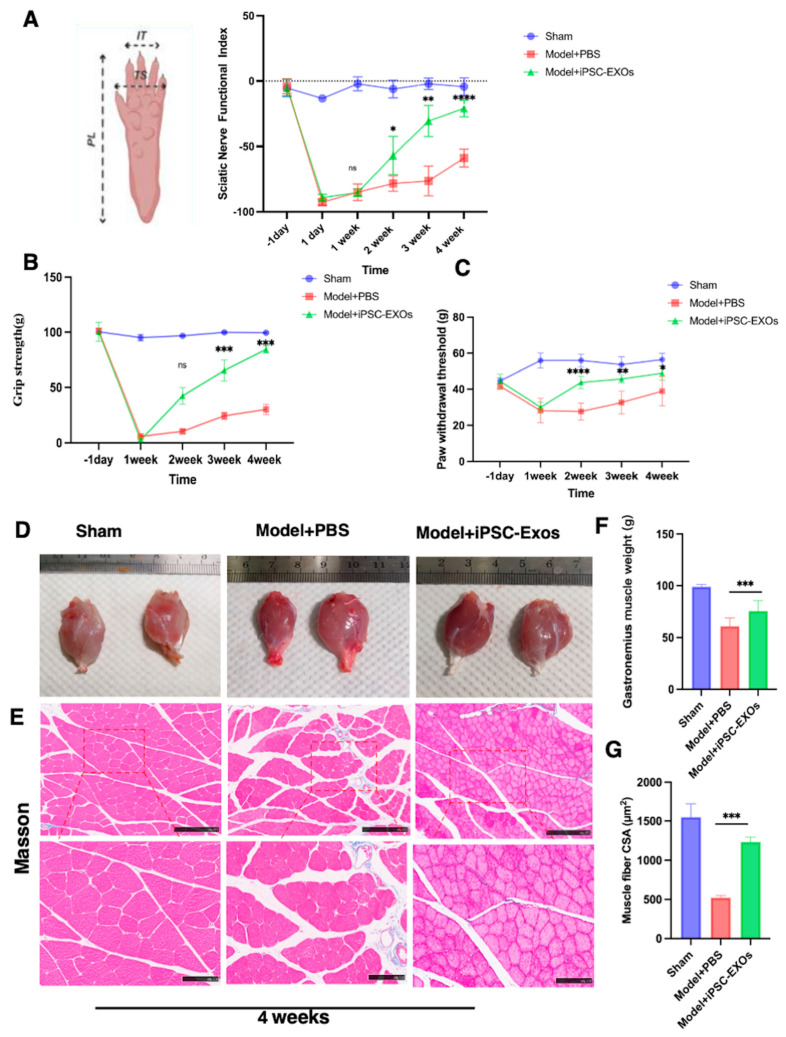
(**A**) Effects of iPSC-Exos on motor and sensory functional outcomes post-crush injury. iPSC-EXOs significantly improved SFI on weeks 2, 3, and 4 compared to the PBS group (n  =  6/group). (**B**) iPSC-Exos significantly improved grip strength on days 14 and 21, 28 post-injury compared to saline (n  =  6/group). (**C**) iPSC-Exoss significantly improved the paw withdrawal threshold as compared to the PBS group postoperative days 3, 7, and 14 (n  =  5/group). (**D**) the evaluation of gastrocnemius muscle atrophy of representative gastrocnemius muscles photographs at 4 weeks post-operative. (**E**) Masson’s trichrome staining of cross-sections of gastrocnemius muscles at 4 weeks post-operative. Scale bars: 250 μm (low magnification images), 100 μm (high magnification images). (**F**) quantitative analysis of wet weight ratios of gastrocnemius muscles (n = 8). (**G**) statistical analysis of each group’s mean cross-sectional area of gastrocnemius muscle fibers (n = 5). Data represent mean ± standard error and were analyzed using one-way ANOVA Tukey’s multiple comparisons test. * *p*  <  0.05, ** *p*  <  0.01, *** *p*  <  0.001, **** *p* < 0.0001, ns: not significant.

To evaluate the efficacy of iPSC-Exos in improving the functional recovery of rats with peripheral nerve crush injuries, the SFI analysis was compared between groups. Walking track patterns were observed preoperative (Figure 6A). Following the sciatic nerve crush one day, SFI values dropped significantly, indicating nerve function loss. By week 1, the iPSC-Exos group showed more functional improvement, and the iPSC-Exos group became evident by week 2, surpassing the Model + PBS (*p* < 0.05), and continued to improve by week 3 *p* < 0.01. On week 4, iPSC-Exos-treated animals demonstrated significant functional recovery compared to the Model + PBS group (*p* < 0.0001). These findings confirm that iPSC-Exos accelerates nerve fiber and sensory and motor function recovery (Figure 6A). Except for SFI analysis, grip strength was evaluated by calculating the average tension force (g) for each group of six rats randomly selected to ensure consistent experimental conditions. Motor function recovery and muscle mass effects of iPSC-Exos were evaluated preoperatively and postoperatively. Grip strength analysis indicated a significant improvement in gripping force recovery over time in the iPSC-EXO-treated group compared to the PBS group starting from week 2 postoperation (Figure 6B). and continuing through week 3 (*p* < 0.001) and week 4 (*p* < 0.001) (Figure 6B). These findings demonstrate that iPSC-Exos significantly accelerates muscle function recovery following injury. The Von Frey filament test is important to evaluate mechanical allodynia, providing a reliable measure of sensory recovery and pain sensitivity during the recovery process after sciatic nerve injury (Figure 6C). Von Frey filament was evaluated preoperatively one day and on weeks 1, 2, 3, and 4 postoperatively to measure the nociceptive threshold to increasing pressure stimuli. In the iPSC-Exos-treated group, significant improvements were noted from week 2 postoperation (*p* < 0.000), with further recovery noted in the third (*p* < 0.01) and fourth (*p* < 0.05) weeks compared to the PBS-treated control group. These findings suggest an enhanced alleviated mechanical allodynia in the iPSC-Exos-treated group (Figure 6C).

### 3.6. iPSC-Exos Prevented Gastrocnemius Muscle Atrophy Following Sciatic Nerve Injury

Following SNI, the gastrocnemius muscle, innervated by the sciatic nerve, becomes atrophy and dysfunction due to prolonged denervation. Denervated muscle loses weight, and muscle fibers shrink as collagen fibers proliferate. Nerve reinnervation may alleviate myoatrophy following implantation. Four weeks post-operation, the bilateral gastrocnemius muscles were harvested and analyzed using Masson’s trichrome staining to examine the histomorphology of the gastrocnemius muscle. The muscle wet weight ratio was higher in the iPSC-Exos group than in the PBS group (*p* < 0.01) (Figure 6D,F). Another parameter of muscle atrophy is decreased muscle fiber diameter) (Figure 6E). Cross-sectional images revealed that muscle fibers in the iPSC-Exos-treated group exhibited improved morphology compared to the PBS-treated group (*p* < 0.001) (Figure 6G). These findings suggest that iPSC-Exos treatment enhances muscle fiber integrity and mass following nerve injury.

### 3.7. iPSC-Exos Promotes Axon Regeneration, Myelination, and Angiogenesis Following Sciatic Nerve Injury

**Figure 7:**
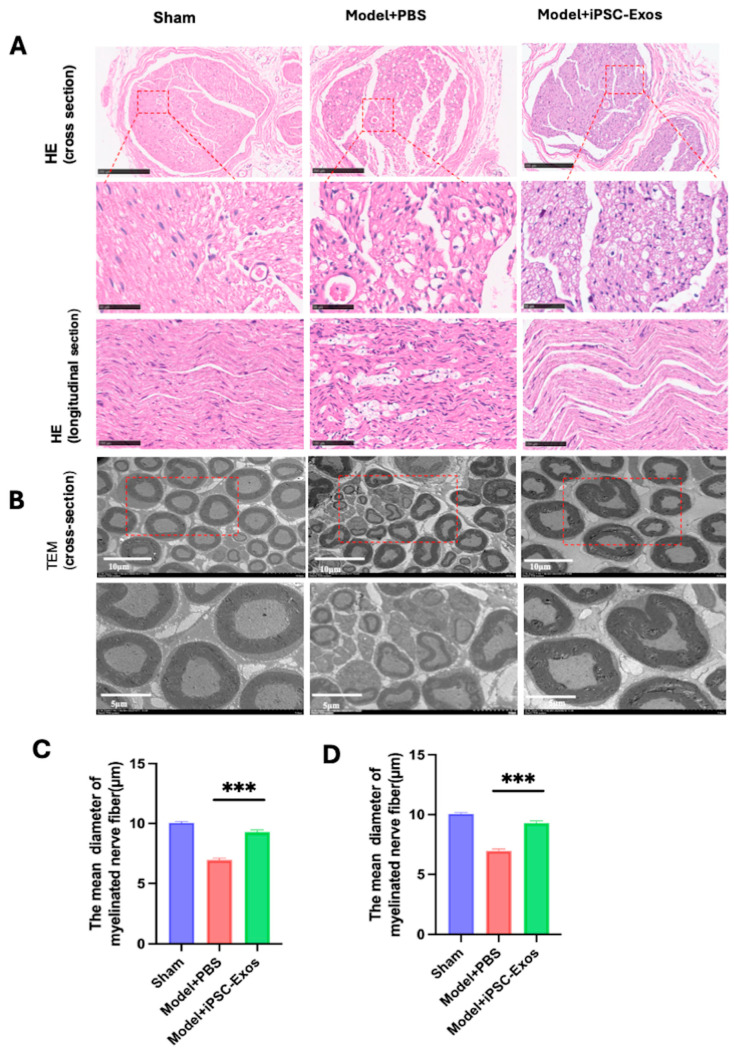
Histological change in injured sciatic nerve with the treatment of iPSC-Exos. (**A**) representative image of HE staining sciatic nerve in cross sections and longitudinal sections in sham, PBS, and iPSC-Exos groups 28 days following the iPSC-Exos treatment of (n = 5). Scale bar, 250 low magnification and 50 μm (higher magnification). (**B**) Representative TEM images of the sciatic nerve in each group. Scale bar, 10 μm (low magnification) and 5 μm (higher magnification). (**C**) Statistical analysis of the mean diameter of axons with myelin. (**D**) Statistical analysis of the mean diameter of axons. Data represent mean ± standard error. *** *p* < 0.001, ns: no significance.

Histological analysis: the H&E-stained sections show histological changes in the sciatic nerve 4 weeks post-injury following iPSC-Exos or PBS treatment. The regenerated nerve fibers in the iPSC-Exos group have a well-organized structure, with many axons and a smaller extracellular space, indicating improved nerve regeneration. Whereas the PBS-treated group has unorganized nerve fibers, increased interfascicular spacing, and obvious edema and vacuolization, indicating weak regeneration (Figure 7A).

Ultrastructural assessment: to further evaluate the effect of iPSC-Exos on myelin, axon regeneration TEM was performed to examine histological changes in the regenerated nerve fibers in all groups (Figure 7B). The histological evaluation, including measurements of myelinated fiber diameter and axon diameter, was conducted at high magnification (Figure 7B). Axons in the iPSC-Exos group exhibited thicker myelin sheaths (Figure 7D). and larger axon diameters (Figure 7C), demonstrating improved remyelination and axonal integrity iPSC-Exos compared to the PBS group.

**Figure 8:**
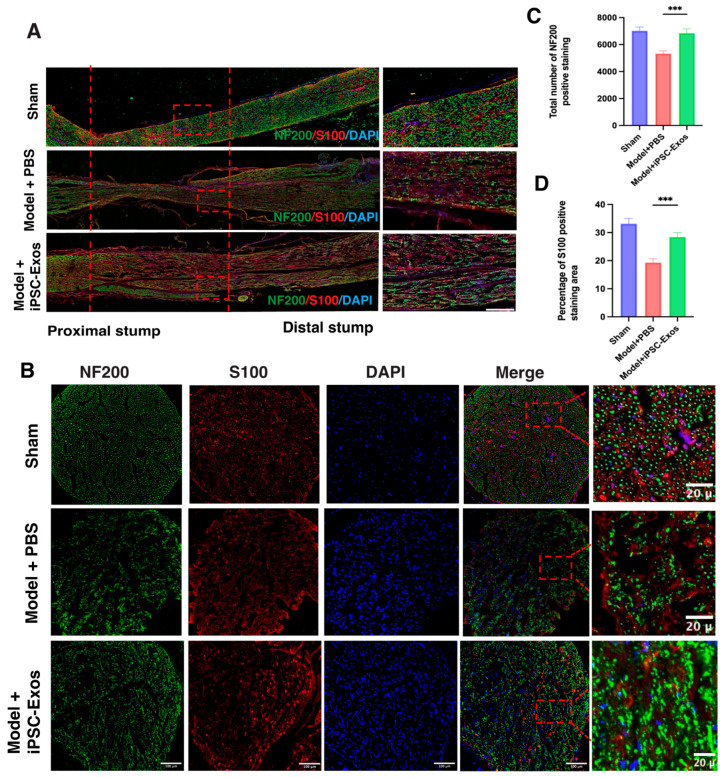
The effects of iPSC-Exos on axon regeneration and myelination were assessed at 2 and 4 weeks postoperatively through immunofluorescence analysis. (**A**) Representative immunofluorescence staining of axons (NF200, green) and myelination markers (S100, red) on longitudinal sections at 2 weeks postoperatively (scale bar = 50 μm). (**B**) Transverse sections axons (NF200, green) and myelination markers (S100, red) 4 weeks postoperatively (scale bar = 100 μm). (**C**,**D**) Statistical analysis quantified the total number of positive stains for NF200, and S100β across all groups. Data are presented as mean ± standard error. SD (n = 5). *** *p* < 0.001.

SC and axon regeneration: To assess the efficacy of iPSC-Exos in nerve regeneration, we performed immunofluorescence staining for the sciatic nerve in five rats for neurofilament-200 (NF200), a marker of neurons forming A-fibers, which plays a crucial role in axon stabilization, maturation, and SCs (S100β) for myelination, which respond to nerve injury by transforming into a repair- related cell phenotype, providing repair signals and spatial guidance that promote axonal regeneration, facilitate target reinnervation and plays a critical role in remyelination (Figure 8A,B), and the process of restoring the myelin sheath around damaged axons. Staining was performed at 2 weeks (n = 3) for longitudinal sections (Figure 8A), and at 4 weeks for cross-sections (n = 5) (Figure 8B). The results showed that the total number of NF200 and S100β were significantly higher in the iPSC-Exos group compared to the PBS-treated group, indicating enhanced axon regeneration and myelination of the injured nerve (Figure 8C,D).

**Figure 9:**
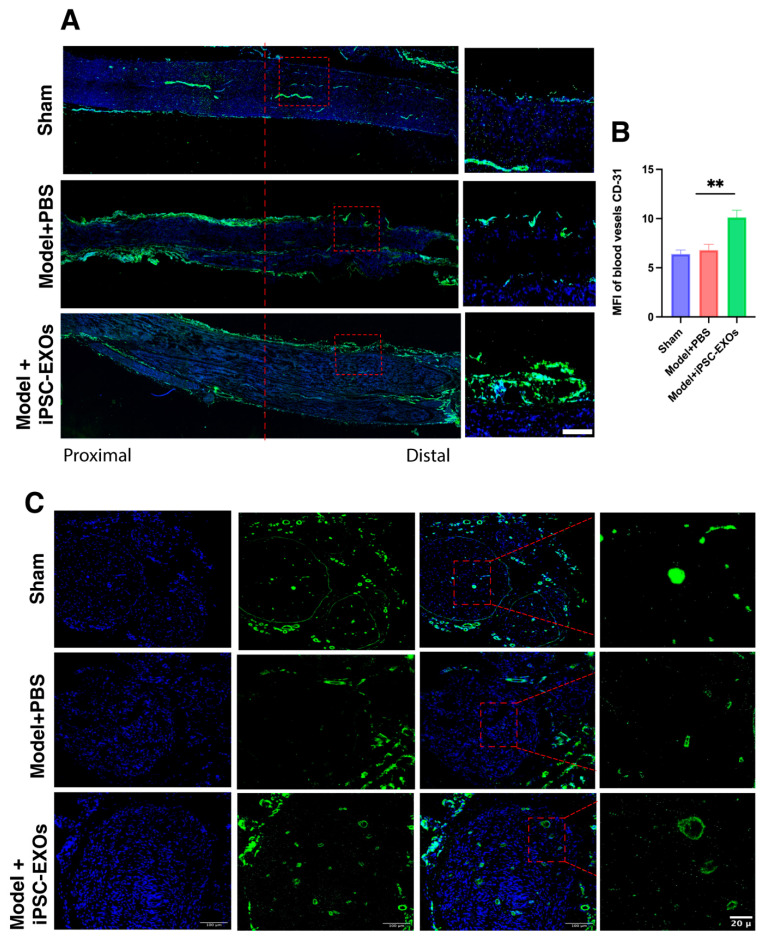
The effects of iPSC-Exos on vascular regeneration were assessed at 2 and 4 weeks postoperatively. (**A**) Immunofluorescence staining of CD31 (endothelial cells, green) was performed on longitudinal sections of neural tissue at 2 weeks postoperatively (scale bar = 50 μm). (**B**) Statistical analysis showed the ratio of the fluorescent area to the total area mean fluorescence intensity (MFI) of CD31 expression, in each group (n = 4 per group). Data are presented as mean ± standard error. ** *p* < 0.01. (**C**) Transverse sections of nerve tissue were analyzed at 4 weeks postoperatively (scale bar = 100 μm).

Angiogenesis Enhancement: to investigate the effect of iPSC-Exos on angiogenesis in the injured nerve, we performed immunofluorescence staining for angiogenesis markers CD31: platelet endothelial cell adhesion molecule-1 (PECAM-1), which is a marker associated with endothelial cells and is involved in angiogenesis. The presence of CD31 correlates with the regeneration of nerve fibers, regeneration [66]. The results of this study revealed in longitudinal sections at 2 weeks (Figure 9A) and cross-sections at 4 weeks (Figure 9B), CD31 expression significantly enhanced across the iPSC-Exos treated group weeks (Figure 9C). These effects may be attributed to the exosomes carrying neurotrophic and vascular endothelial growth factors, including VEGF, FGF2, TGF-β, NRF2, BDNF, and angiopoietins [67], which communally promote angiogenesis, axon regeneration, myelination, and sensory and motor function recovery while stabilizing vasculature in injured nerves. These factors create an optimal microenvironment for nerve repair and tissue regeneration. iPSC-Exos markedly enhanced axonal regeneration, myelination, and functional recovery in injured sciatic nerves.

### 3.8. iPSC-Exos Boost Nerve Repair by Modulating Key Molecular Pathways: A Transcriptomic Analysis

**Figure 10:**
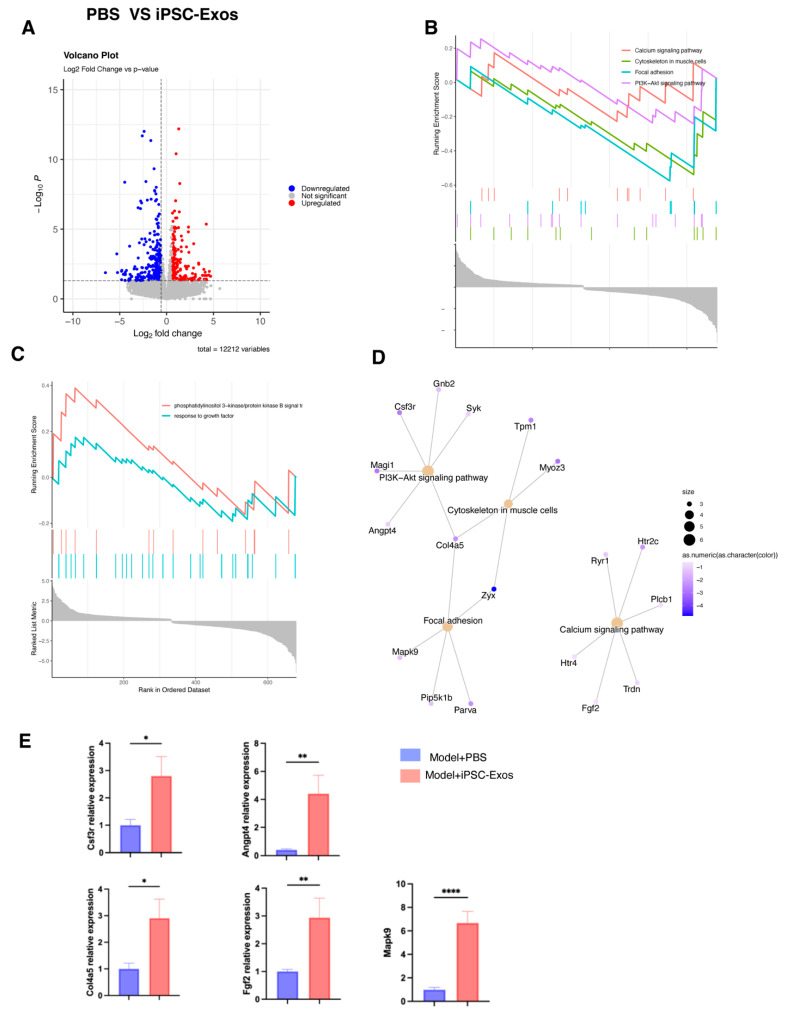
(**A**) Volcano plot of genes with differential expression in the PBS and iPSC-Exos groups, revealing significantly upregulated genes (red) and downregulated (blue). (**B**) Gene Set Enrichment Analysis (GSEA) of KEGG pathways related to nerve repair, including PI3K-AKT signaling, focal adhesion, cytoskeleton regulation, and calcium signaling. (**C**) Gene Set Enrichment Analysis (GSEA) of Gene Ontology (GO) terms demonstrates enrichment in biological processes involved in nerve regeneration, such as phosphatidylinositol signaling and growth factor responses. (**D**) Cluster Network (CNET) plot showing the link between nerve repair-related genes and the associated KEGG pathways. (**E**) Validation of RNA-seq results using PCR analysis of specified mRNAs, confirming differential expression in the PBS and iPSC-Exos treatment groups. Data are presented as means ± SEM, and statistical significance was determined using an independent samples *t*-test (* *p* < 0.05, ** *p* < 0.01, **** *p* < 0.0001).

RNA sequencing demonstrated different gene expression profiles between the model + PBS group and the model + iPSC-Exos treated group. The differential expression analysis determined significantly upregulated or downregulated genes, as shown in the volcano plot. Upregulated genes are represented in red, while those downregulated are represented in blue, indicating potential candidates for further exploration of the Pathway enrichment analysis (Figure 10A). KEGG pathway analysis revealed notable activation of pathways such as PI3K-AKT signaling, Focal Adhesion, Cytoskeleton in Muscle Cells, and Calcium Signaling Pathway, underlining their involvement in boosting cellular survival, proliferation, and repair mechanisms (Figure 10B). A complementary Gene Ontology (GO) enrichment study revealed processes such as PI3K/AKT signal transduction and response to growth factor, which help clarify the molecular pathways activated by iPSC-Exo treatment (Figure 10C). Furthermore. Genes like Csf3r, Mapk9, and col4a5 Angptl4 had significantly higher expression levels in the iPSC-Exosome group compared to the PBS group (Figure 10D). To confirm the RNA-seq results, qPCR was performed for selected differentially expressed genes related to nerve regeneration. The qPCR results were consistent with the RNA-seq data, showing significant upregulation of these key genes in the iPSC-Exos-treated group (Figure 10E). This alignment validated the accuracy and reliability of the transcriptomic analysis and provided further proof that iPSC-Exos modulates vital biological pathways, including angiogenesis, nerve regeneration, and tissue remodeling, to promote effective nerve repair and functional recovery. Overall, the results suggest that iPSC-Exos holds therapeutic potential in nerve repair and advancing applications in regenerative medicine.

## 4. Discussion

PNI is a complex condition that commonly leads to motor and sensory impairment in patients [68]. Currently, the prognosis for PNI is poor, and there is an urgent need for effective treatment for PNI [69]. Stem cell-based therapy has convenient, promising benefits for PNI by boosting nerve regeneration, as demonstrated by enhanced axonal regeneration, myelin formation, new vessel formation, and subsequent sensory and motor neuron recovery [70]. These benefits are accompanied by significant improvements in motor function [70]. Furthermore, cell transplantation creates an optimal environment for damaged nerves to recover, proliferate, and regenerate while exhibiting anti-inflammatory by secreting neurotrophic factors (NTFs) and EVs, known as exosomes [71]. Exosomes are crucial bioactive components derived from various MSCs. Besides, Exosomes avoid some common issues associated with cell therapies, such as tumorigenicity, vascular blockage, and short shelf life [72]. While iPSCs are shown to be non-tumorigenic and to have neuroprotective and axonotropic capacities, the therapeutic application of iPSCs is limited due to unknown long-term effects [73]. iPSC-Exos presents a promising way to address the limitations of iPSCs in nerve regeneration [74]. These nanosized extracellular vesicles facilitate intercellular communication and deliver bioactive molecules that promote the regenerative processes in neural tissues [75]. Their properties, such as stability, immune tolerance, and ability to pass through the blood-brain barrier, make them superior to traditional stem cell therapies [76].

The findings of our study indicate the effective generation of iPSCs from PBMCs using the SeV vector. This method is advantageous as it is non-integrating, which reduces the possibility of genetic alterations [77]. The iPSC colonies exhibited ESC-like morphology and expressed vital pluripotency markers like NANOG, OCT4, SOX2, and SSEA4, consistent with previous results [78]. PCR analysis revealed the absence of remaining SeV components, indicating that the reprogramming technique does not leave residual viral elements, confirming the safety of this reprogramming technique [79]. Extended culture has a significant effect on cell karyotype, resulting in instability of chromosomes and potentially serious consequences. Karyotypic changes during cell culture frequently appear as aneuploidy, deletions, and duplications, which are mainly caused by mechanisms such as DNA repair deficits, telomere attrition, and spindle assembly defects [80]. The results of the karyotype experiment in passages 10 and 35 showed a normal 46, XY karyotype, and genetic stability through extended culture. In vivo differentiation experiments confirmed the formation of tissues from all three germ layers, indicating the pluripotency of the iPSCs. These findings demonstrate the efficacy and reliability of SeV vectors in transforming PBMCs into therapeutically effective iPSCs. Despite the increased interest in iPSC-Exos research on their safety and toxicity is lacking, and there is an urgent need to estimate the safety of Exosomes. Our findings demonstrated that iPSC-Exos had no adverse impacts on the physical results of the rats this included rats’ body weight being monitored as an indicator of growth trends. The CBC analysis in this study showed no significant differences between groups, consistent with results from the previous study [81]. However, group variations, including values exceeding reference ranges, may result from biological variability, experimental conditions, and strain-specific references [82]. iPSC-Exos are safe, as they did not impact key blood indicators, these findings support previous research using C57BL/6J mice injected with 6 × 10^10^ Exosome particles, as a therapeutic dose, no abnormalities were observed in the treated mice [83]. In another study, an intravenous injection of 3.85 × 10^12^ Exosome particles was administered to cynomolgus monkeys [81]. Observations showed no significant behavioral changes, neurological abnormalities, or mortality [81]. In addition, our findings indicate that iPSC-Exos injections are safe and non-toxic in SD rats, as indicated by normal histology and organ weights, with no noticeable abnormalities in major organs like the heart, liver, spleen, lungs, and kidneys histopathology. These findings are in line with previous studies conducted by Hu, Wang et al., 2024 [81]. However, many issues remain unresolved before exosomes can be used in clinical therapy. For instance, studies report that exosomes from bone marrow MSCs may promote tumor growth [84]. In addition, issues remain about the standardization of exosome production. This highlights the need to confirm the safety of exosomes from other sources and complete safety investigations before clinical applications can be established [85,86] as well as various administration routes [87]. Recently, many researchers have demonstrated the potential of MSCs-Exos in facilitating PNI repair and promoting axonal regeneration through the stimulation of endogenous SC proliferation and migration [86,88]. The present study utilized the conventional rat sciatic nerve injury (SNI) model, specifically a crush injury classified as sunderland grade III damage [89]. In this type of injury, nerve continuity depends on an intact epineurium. While the axons, endoneurium, and perineurium are all damaged. Compared with discontinuous injury, this type produces less extensive damage and allows for faster recovery, reducing the observation period and minimizing the impact of surgical repair techniques [68]. This procedure helps to avoid potential interference from surgical repair on functional recovery outcomes and optimize the experiment’s comparability and reliability [90]. In this study, we demonstrated the significant neuro-regenerative effects of iPSC-Exos and their ability to enhance both motor and sensory functions. The grip strength and SFI analyses, widely accepted as standard methods for evaluating nerve recovery, showed that iPSC-Exos treatment significantly accelerates functional and muscle strength recovery in rats with SNI. These findings align with previous studies, supporting the efficacy of iPSC-Exos in accelerating motor function, sensory function, and muscle strength recovery over time [91,92]. Furthermore, this study showed that iPSC-Exos treatment significantly affects the wet-weight ratio and gastrocnemius muscle fiber morphology by enhancing nerve regeneration and subsequent muscle recovery. The bioactive cargo (neurotrophic factors, microRNAs, structural proteins, and immunomodulatory cytokines) of iPSC-Exos in nerve regeneration is attributed to their therapeutic potential, which coordinates a complex regenerative response following peripheral nerve injury [93,94]. Our results show that iPSC-Exos promotes functional recovery, axon regeneration, myelination, and angiogenesis and modifies the neuroinflammatory milieu. One of the primary mechanisms by which iPSC-Exos facilitates nerve repair and axon growth is delivering growth-promoting proteins, and miRNAs facilitate cell communication, which is crucial for axonal guidance and growth [95]. In our study, the significant upregulation of NF200, a marker of axonal regeneration and growth, suggests enhanced axon regeneration following iPSC-Exos treatment. In addition to axonal regrowth, effective remyelination is essential for functional nerve regeneration. Our results indicate that iPSC-iPSC-Exos promotes SC proliferation and myelin formation, which is confirmed by an increase in *S100B*, *PMP22*, and *MPZ*. These molecules are essential for SC function and myelin compaction, ensuring the structural integrity of regenerating axons. Additionally, miR-29a, a microRNA known to regulate myelin gene expression, has been implicated in the stabilization of myelin sheaths, indicating the role the role of iPSC-Exos in remyelination [96]. The significant improvement in SFI and grip strength in our study suggests that enhanced myelination contributes to the functional restoration observed in the iPSC-Exos group. Vascularization plays a vital role in peripheral nerve regeneration, as re-establishing blood vessels ensures sufficient nutrient and oxygen supply to injured tissues. The increased expression of *Angpt4*, *VEGFA*, and CD31 in our finding suggests that iPSC-Exos promotes angiogenesis and angiogenesis associated with extracellular matrix remodeling. Furthermore, miR-126, a key pro-angiogenic microRNA, has been shown to enhance endothelial cell function and blood vessel formation, supporting our finding that iPSC-Exos creates a favorable microenvironment for nerve regeneration by enhancing vascular support [93]. The immune response to nerve injury plays a dual role, with early inflammation supporting debris removal and constant inflammation impairing regeneration. Our findings suggest that iPSC-Exos can reduce neuroinflammation by delivering anti-inflammatory cytokines (IL-10). *IL-10, TGF-β*, and microRNAs (miR-146a) molecules reduce NF-κB, converting the immune response to a pro-regenerative state and attenuating pain, indicating their potential use in pain management for nerve injury recovery [94,95]. *Col4a5* is increased in SC after nerve injury and has a critical role in ensuring the regenerating axons select their original pathways. On the other hand, *Col4a5* binds to axon repellents such as Slit and Netrin, which are vital for axonal guidance. Upregulating *Col4a5* after injury is critical for the extracellular matrix (ECM) role in providing a conducive environment for axonal growth and guidance [97,98]. Pathway enrichment analysis of KEGG demonstrated the activation of pathways, such as PI3K/AKT signaling, vital for neuronal survival, axonal regeneration, promoting cell proliferation, and inhibiting apoptosis. These results are consistent with a previous study using endothelial cell-derived exosomes (EC-Exo) to promote axonal regeneration and myelination by activation of the PI3K/AKT signaling pathway is essential for shifting SC transformation into a repair-related phenotype [99]. Further, iPSC-Exos promotes nerve repair through focal adhesion mechanisms by enhancing the interaction between SC and the ECM, enhancing focal adhesion formation, which is essential for cell migration and nerve regeneration [100]. iPSC-Exos plays a significant role in modulating calcium signaling pathways during nerve regeneration [99]. Their interactions with SCs and endothelial cells have been demonstrated to enhance SC activation and function. Heatmap analysis revealed that the iPSC-Exosome group expressed more important genes like *Gdnf*, *Fgf2*, and *Csf3r*. This well-known neurotrophic factor has been shown to improve neuronal survival and regeneration after PNI [101].

Together, the results of this study demonstrate the therapeutic potential of iPSC-Exos in promoting nerve regeneration without adverse effects on body weight, blood parameters, or organ histology. Functionally, iPSC-Exos promotes motor recovery, muscle regeneration, and myelination, as indicated by improved grip strength, SFI, and upregulation of key neural markers. iPSC-Exos also enhanced angiogenesis and reduced neuroinflammation, creating a pro-regenerative environment. Furthermore, they activated the PI3K/AKT pathway, supporting axonal growth and Schwann cell function.

### Limitations

This study demonstrates the therapeutic effects of iPSC-Exos in the SNI model; however, some limitations remain. First, the effects of iPSC-Exos on nerve repair were not compared with cell transplantation or other types of exosomes. Second, the safety and efficacy of iPSC-Exos at different doses were not investigated. Third, to examine potential sex differences, future studies should include female rats. Furthermore, the molecular mechanisms and long-term effects of iPSC-Exos on regeneration outcomes require extensive investigation. Within these limitations, the achieved results provide valuable insights for clinical practice.

## 5. Conclusions

The evidence obtained in this study indicated that iPSC-Exos significantly enhances functional recovery, attenuates pain, and accelerates sciatic nerve regeneration by increasing nerve fiber myelination, axonal growth, angiogenesis, extracellular matrix integrity while establishing an optimal environment for long-term peripheral nerve regeneration and functional recovery. Furthermore, iPSC-Exos activated key pathways like PI3K-AKT and focal adhesion signaling, critical for neuronal survival, and axonal regeneration. These findings provide the foundation for future investigation into the clinical application of iPSC-Exos as a novel therapeutic method for peripheral nerve regeneration.

## Figures and Tables

**Table 1:** Forward and reverse primer sequences for target genes.

Gene	Direction	Primer Sequence
*Akt1*	Forward	GTGGCAAGATGTGTATGAG
	Reverse	CTGGCTGAGTAGGAGAAC
*PMP22*	Forward	TCGCGGTGCTAGTGTTGC
	Reverse	GACAGGACGCTGAAGATGACA
*IL-10*	Forward	CAGTCAGCCAGACCCACAT
	Reverse	GCTCCACTGCCTTGCTTT
*S100b*	Forward	TTGCCCTCATTGATGTCTTCCA
	Reverse	TCTGCCTTGATTCTTACAGGTGAC
*MBP*	Forward	CCC CAG CTA AAT CTG CTG AG
	Reverse	CCC CAG CTA AAT CTG CTG AG
*MPZ*	Forward	CCT TCA AAT ATG CCT GGG T
	Reverse	CAG CAC AGT CAG CTT GAG AG
*BDNF*	Forward	CGTGGGGAGCTGAGCGTGTG
	Reverse	GCCCCTGCAGCCTTCCTTC
*Col4a5*	Forward	GATCTCCAGGTGACCAAGGA
	Reverse	CCTGAAATGCCAGTTCCAA
*FGF2*	Forward	ACCCGGCCACTTCAAGG
	Reverse	GATGCGCAGGAAGAAGCC
*ANGPT4*	Forward	GCAAGGCACCACCTAACAGA
	Reverse	GATGGACTGCTCCAGCTTCA
*CSF3R*	Forward	GTTCTGCTGCAAGCAAAGCA
	Reverse	GCAGCTGGAAGGTTTCCTCT
*GAPDH*	Forward	TGC TGA GTA TGT CGT GGA G
	Reverse	GTC TTC TGA GTG GCA GTG AT

**Table 2:** Hematological Parameters in Sham, Model + PBS, and Model + iPSC-Exos Groups.

Variables	Sham		Model + PBS		Model + iPCS-Exos		Reference Range
	Mean	SD	Mean	SD	Mean	SD	
WBC	11.47	3.43	11.72	3.31	9.18	1.37	8.9–13.9
RBC	7.29	13.88	7.02	1.65	8.11	0.27	9.6–11.3
HGB	143.75	0.67	135.83	37.05	159	6.68	137–160
HCT	38.9	4.60	38.36	8.74	42.55	1.72	42.2–49.5
MCV	53.25	0.23	54.88	2.09	52.45	0.75	43.1–44.1
MCH	19.72	1.188	19.15	1.13	19.57	0.23	13.6–15.3
MCHC	370.7	11.87	350	31.4	373	3.74	317–330.4
PLT	372.5	182	360.1	281.4	422.25	195.93	929–1089.5
Lym%	61.27	8.2	67.4	7.54	68.97	10.40	51.5–66.5
Neu%	30.5	7.86	23.6	7.55	19.87	6.89	10–30
Mon%	3.5	1.56	4.72	2.163	7.55	2.63	2–6
Eos%	4.5	1.47	3.85	1.33	3.42	1.03	0.5–4.5
Bas%	0.675	0.63	1.05	0.59	0.17	0.09	0.00–0.089
Neu#	3.41	1.18	2.8	1.29	1.88	0.94	1.83–3.6
Lym#	7.1	2.53	7.85	2.16	6.25	0.66	6.3–10.6
Mon#	0.36	0.24	0.49	0.30	0.71	0.36	0.1–0.6
Eos#	0.51	0.05	0.44	0.16	0.31	0.12	0.158–2.01
Bas#	0.07	0.05	0.125	0.08	0.017	0.009	0.0–0.67
RDW-SD	28.5	1.42	29.5	1.336	27.27	0.65	38–50
RDW-CV	14.4	0.46	14.45	0.45	14.1	0.31	13.0–18.5
MPV	8.3	0.52	7.43	0.37	7.27	0.20	13.8–15.4
PLCR	18.82	4.5	12.36	2.72	11.6	1.49	20–35
PDW	15.3	0.09	15.15	0.23	15	0.081	-
PCT	0.31	0.14	0.26	0.201	0.3075	0.138	-
PCT	0.31	0.14	0.26	0.201	0.3075	0.138	-

## Data Availability

The data supporting the findings of this study are available from the corresponding author upon reasonable request. All RNA-seq data supporting this study are available online in the Gene Expression Omnibus (GEO) database under the accession number GSE286150. The data is scheduled to be made publicly available on 8 January 2026.

## Abbreviations

AKT1	AKT Serine/Threonine Kinase 1
ANOVA	Analysis of Variance
BDNF	Brain-Derived Neurotrophic Factor
CD31	Cluster of Differentiation 31 (Platelet Endothelial Cell Adhesion Molecule)
IF	Immunofluorescence
ECM	Extracellular matrix
IL-10	Interleukin-10
iPSCs	Induced Pluripotent Stem Cells
iPSCs-Exos	Induced Pluripotent Stem Cells-Derived Exosomes
KEGG	Kyoto Encyclopedia of Genes and Genomes
MBP	Myelin Basic Protein
MPZ	Myelin Protein Zero
MSCs	Mesenchymal Stem Cells
NF200	Neurofilament 200
NTA	Nanoparticle Tracking Analysis
PMP22	Peripheral Myelin Protein 22
PNI	Peripheral Nerve Injury
SCs	Schwann Cells
SD	Standard Deviation
SEM	Mean ± Standard Error of the Mean
S100β	S100 Calcium-Binding Protein Beta
SFI	Sciatic Nerve Functional Index
SNI	Sciatic Nerve Injury
TEM	Transmission Electron Microscopy
VEGFA	Vascular Endothelial Growth Factor A
WT	Withdrawal Threshold
